# The Predictive Role of the Systemic Inflammation Response Index in the Prognosis of Hepatitis B Virus-Related Acute-on-Chronic Liver Failure: A Multicenter Study

**DOI:** 10.3390/healthcare13172199

**Published:** 2025-09-02

**Authors:** Jing Yuan, Jing Chen, Haibin Su, Yu Chen, Tao Han, Tao Chen, Xiaoyan Liu, Qi Wang, Pengbin Gao, Jinjun Chen, Jingjing Tong, Chen Li, Jinhua Hu

**Affiliations:** 1 Medical School of Chinese People’s Liberation Army (PLA), Beijing 100853, China; jyuan3012025@126.com; 2 Senior Department of Hepatology, The Fifth Medical Center of PLA General Hospital, Beijing 100039, China; chenj19939327@163.com (J.C.); suhaibin302@163.com (H.S.); dwyxzxlxy@163.com (X.L.); gemini_lee525@126.com (C.L.); 3 Senior Department of Hepatology, Beijing Youan Hospital Affiliated to Capital Medical University, Beijing 100069, China; chybeyond@163.com; 4 Department of Gastroenterology and Hepatology, Tianjin Union Medical Center Affiliated to Nankai University, Tianjin 300121, China; hantaomd@126.com; 5 Department of Infectious Diseases, Tongji Hospital of Tongji Medical College, Wuhan 430030, China; chentao_tjh@vip.sina.com; 6 National Center for Infectious Diseases, Beijing Ditan Hospital, Capital Medical University, Beijing 100015, China; wangqidl04@126.com; 7 Department of Infectious Diseases, Shijiazhuang Fifth Hospital, Shijiazhuang 050024, China; gaopbky@163.com; 8 Hepatology Unit, Department of Infectious Diseases, Nanfang Hospital, Southern Medical University, Guangzhou 510515, China; chjj@smu.edu.cn; 9 Department of Infectious Diseases, China-Japan Friendship Hospital, Beijing 100029, China; tongjingjingcat@163.com

**Keywords:** systemic inflammation response index, acute-on-chronic liver failure, dynamic changes, prognosis

## Abstract

Background/Objectives: The prognosis of patients with hepatitis B virus-related acute-on-chronic liver failure (HBV-ACLF) is significantly affected by inflammatory state and immune dysregulation. The systemic inflammatory response index (SIRI), which reflects neutrophil, monocyte, and lymphocyte dynamics, has emerged as a potential marker of immune-inflammatory status. However, its role in predicting HBV-ACLF outcomes remains unclear. This research aims to elucidate the prognostic value of SIRI and its dynamic changes combined with disease severity scores in predicting the outcomes of HBV-ACLF. Methods: The study included HBV-ACLF patients enrolled in a multicenter clinical study between July 2019 and April 2024. Based on 90-day outcomes, the participants were categorized into survival and death groups. Clinical data and SIRI values were collected on days 0 (baseline), 3, 7, and 14. Independent prognostic factors were identified using Cox regression and least absolute shrinkage and selection operator (LASSO) analysis. The predictive value of dynamic SIRI changes combined with disease severity scores was evaluated using receiver operating characteristic (ROC) curves. Results: A total of 153 patients with HBV-ACLF were analyzed, including 104 in the survival group and 49 in the death group. SIRI values were significantly lower in the survival group than in the death group across all time points. Multivariate Cox regression analysis identified that an increased ΔSIRI at day 3 (ΔSIRI3), a higher MELD score, and a lower albumin level were independently associated with increased 90-day mortality. The combination of SIRI on day three (SIRI3) and MELD-Na score on day three (MELD-Na3) demonstrated the highest predictive performance, with an AUC of 0.817 (95% CI: 0.750–0.883). Conclusions: The combination of the SIRI and MELD-Na score on day three provides a strong predictive value for the short-term prognosis of HBV-ACLF, highlighting its potential utility in early prognostic evaluation.

## 1. Introduction

Acute-on-chronic liver failure (ACLF) is characterized by hepatic and/or systemic organ dysfunction with high short-term mortality [1,2]. It involves acute deterioration of liver function against a background of chronic liver disease, often leading to multiple organ failure. In China, hepatitis B virus-related ACLF (HBV-ACLF) is the predominant form of ACLF [3,4,5]. Chronic liver damage caused by hepatitis B virus infection, including fibrosis and cirrhosis, provides the pathological basis for ACLF development. Acute triggers, including the active replication of the hepatitis B virus, alcohol consumption, and infections, can cause extensive necrosis of hepatocytes and rapid deterioration of liver function [5,6]. Therefore, HBV acts not only as the underlying cause of chronic liver disease but also as the trigger for the acute onset of HBV-ACLF [7].

HBV infection can trigger systemic inflammatory response syndrome (SIRS), leading to multiple organ failure involving the liver, kidneys, brain, lungs, coagulation abnormalities, and circulatory system [8,9]. In ACLF, systemic inflammation serves as the major driver of organ dysfunction through three main mechanisms: (1) immune-mediated injury, such as neutrophil extracellular traps (NETs) that cause oxidative stress and microcirculatory dysfunction; (2) metabolic dysregulation, in which energy resources are diverted to sustain immune activation, leading to mitochondrial dysfunction; (3) interaction with organ-specific pathways, such as proinflammatory cytokines that increase blood–brain barrier permeability and activate microglia, thereby aggravating hepatic encephalopathy [5,6,10]. In addition to liver failure, ACLF frequently affects extrahepatic organs. Hemodynamic changes, including splanchnic vasodilation, decreased systemic vascular resistance, and impaired cardiac output, contribute to tissue hypoperfusion and organ injury. Tumor necrosis factor-α (TNF-α)-induced nitric oxide signaling contributes to cardiac dysfunction [11], while chemokine-mediated inflammatory responses negatively affect pulmonary function [12]. Renal injury often results from renal vasoconstriction and reduced glomerular filtration [13], while neurological impairment is linked to hyperammonemia and cerebral edema [14]. These organ failures and SIRS establish a vicious cycle: SIRS induces vascular endothelial injury and impairs organ perfusion, while organ failure, particularly of the liver and kidneys, exacerbates inflammation by reducing clearance of cytokine release. This bidirectional interaction accelerates disease progression and worsens prognosis in ACLF patients [15].

In HBV-ACLF, pathogen-associated molecular patterns (PAMPs) are released due to HBV infection, intestinal barrier dysfunction, bacterial translocation, and increased susceptibility to microbial invasion. When PAMPs bind to pattern recognition receptors (PRRs), they trigger innate immune activation, resulting in cell death and tissue damage in both hepatic parenchymal and non-parenchymal cells. Furthermore, damage-associated molecular patterns (DAMPs) stimulate the release of pro-inflammatory cytokines such as IL-1β, IL-6, and IL-8, contributing to cytokine storm and SIRS [16,17]. In HBV-ACLF patients, monocytes and macrophages demonstrate reduced HLA-DR expression and metabolic dysfunction, resulting in impaired antigen presentation, phagocytic activity, and oxidative burst [18,19]. Neutrophils display multiple functional defects, including impaired phagocytosis, chemotaxis, degranulation, and NETs formation, thereby increasing susceptibility to infections [20]. Peripheral natural killer (NK) cells are decreased due to enhanced apoptosis and migration, while intrahepatic NK cell accumulation contributes to liver injury [21]. Interferon-γ (IFN-γ), primarily produced by NK and T cells, plays a dual role in antiviral immunity and immunopathology in HBV-ACLF [22]. In CD4^+^ T cells, the upregulation of B- and T-lymphocyte attenuator (BTLA) via IL-6 and TNF signaling suppresses their activation and cytokine production, correlating with disease severity [23]. The imbalance of pro- and anti-inflammatory cytokines, including TNF-α, IL-6, IL-10, and IFN-γ and the resulting cytokine storm drive organ dysfunction and disease progression in ACLF [24,25,26].

Liver transplantation is considered one of the most effective treatments for ACLF. Nevertheless, the limited availability of liver donors and high costs have restricted its widespread clinical application. Meanwhile, no effective medical therapies are currently available. Therefore, early evaluation of the short-term prognosis in ACLF patients is crucial for guiding clinical treatment. Although there are several clinical prognostic models for ACLF, such as the Child–Turcotte–Pugh (CTP), model for end-stage liver disease (MELD), the model for end-stage liver disease combined with serum sodium (MELD-Na), and the chronic liver failure combined with organ failure score (CLIF-C OFs), their clinical utility is limited due to complex calculations and the inclusion of multiple variables [27,28,29]. Additionally, most of these models were developed in Western countries, where alcohol-related liver disease is the predominant etiology of ACLF, while, in China, HBV infection is the primary cause. Consequently, there is an urgent need to identify more accessible and reliable prognostic markers for HBV-ACLF patients in China.

The systemic inflammatory response has been proposed as one of the mechanisms for ACLF [30]. Infection, cytokine release, and immune dysfunction can trigger or exacerbate a strong inflammatory response, resulting in impaired systemic circulation, organ dysfunction, and poor clinical outcomes [25,31]. Therefore, it is essential to incorporate inflammation-related indicators in the prognostic assessments. The initiation and progression of ACLF are closely associated with systemic inflammation, with neutrophils, monocytes, and lymphocytes serving as key immune effector cells [32,33,34]. The neutrophil-to-lymphocyte ratio (NLR), platelet-to-lymphocyte ratio (PLR), and red cell distribution width (RDW) have been demonstrated to predict the short-term prognosis of ACLF patients [10,35]. Nonetheless, most of the above inflammatory indicators only consist of one or two hematological parameters, which cannot fully reflect the systemic inflammatory and immune status, thereby limiting their prognostic value. The systemic inflammation response index (SIRI) is an innovative index for assessing inflammation derived from these immune cells, reflecting the balance between inflammation and immune status [36,37]. SIRI was initially proposed by Qi et al. to predict the prognosis of pancreatic cancer patients undergoing chemotherapy in 2016, demonstrating excellent predictive efficacy [36]. Recent studies have demonstrated strong associations between SIRI and prognosis in various cancers and cardiovascular diseases [38,39,40]. However, the prognostic significance of SIRI fluctuations in ACLF remains unclear. This research aimed to evaluate the prognostic value of dynamic changes in SIRI for predicting the outcomes of HBV-ACLF (MELD score 16–32). Furthermore, the use of a multicenter cohort and rigorous statistical methods (LASSO + Cox + ROC) provides a novel and robust approach to early prognostic assessment in HBV-ACLF patients.

## 2. Materials and Methods

### 2.1. Study Design

This study was designed as a prospective, multicenter clinical study. A total of 153 HBV-ACLF patients meeting the inclusion and exclusion criteria were enrolled from seven hospitals in China between July 2019 and April 2024: (1) The Fifth Medical Centre of the General Hospital of the People’s Liberation Army; (2) Beijing Youan Hospital of Capital Medical University; (3) Beijing Ditan Hospital Capital Medical University; (4) Tianjin Third Central Hospital; (5) Shijiazhuang Fifth Hospital; (6) Tongji Hospital of Tongji Medical University; and (7) Nanfang Hospital of Southern Medical University. This research received approval from the leading ethics committee, the Fifth Medical Center of the PLA General Hospital (2020041D) and was further reviewed and approved by the ethics committees of all participating centers. The study adhered to the ethical guidelines of the Declaration of Helsinki, 1964.

### 2.2. Inclusion Criteria

All participants, infected with the hepatitis B virus, met the diagnostic criteria for ACLF established by the Asia Pacific Association for the Study of the Liver (APASL) [41].

The inclusion criteria were as follows:

(1) Individuals aged 18–65;

(2) Clinical diagnosis of HBV-ACLF, as determined by:

- Chronic hepatitis B infection based on persistent HBsAg or HBV-DNA positivity for over 6 months;
- Serum total bilirubin ≥ 5 mg/dL (85 μmol/L);
- INR ≥ 1.5 or PTA < 40%;
- Development of ascitic or hepatic encephalopathy within a month;
- MELD score ranging from 16 to 32.

To ensure diagnostic accuracy and adherence to APASL criteria, all cases were independently reviewed and confirmed by two senior hepatologists prior to inclusion.

### 2.3. Exclusion Criteria

(1) Co-infection with other hepatotropic viruses;

(2) Presence of acute kidney injury (AKI);

(3) Severe extrahepatic organ dysfunction, including disease of the heart, brain, lungs, kidneys, or other organs diseases, especially uncontrolled bacterial infections such as sepsis or septic shock;

(4) Bone marrow aplasia;

(5) Malignant neoplasms;

(6) Pregnant or lactating women.

In addition, patients with Grade III–IV hepatic encephalopathy were not enrolled, as these cases are widely recognized in international guidelines as indicative of advanced hepatic failure and are frequently associated with a poor short-term prognosis or the need for urgent liver transplantation. To minimize prognostic bias associated with end-stage disease and ensure cohort homogeneity in evaluating outcomes of medically managed HBV-ACLF patients without liver transplantation, only patients with Grade I–II hepatic encephalopathy were included.

### 2.4. Treatment Protocols

All patients received standardized treatment according to national liver failure guidelines [42]. They received antiviral therapy, improvements in liver function, and general medical treatment. Some went through artificial liver support therapy.

### 2.5. Sample Size Estimation

Given the exploratory nature of this study and the lack of previously published data on the SIRI value measured on day 3 in patients with HBV-ACLF, a formal a priori sample size calculation was not feasible at the study design stage. Therefore, a post hoc sample size estimation was performed using actual data from our prospective cohort to assess whether the sample size was adequate for detecting clinically meaningful differences. On Day 3, the mean SIRI values in the survival and death groups were 2.28 and 5.34, respectively, with standard deviations of 2.39 and 6.04. The calculated effect size (Cohen’s d) was approximately 0.67, which indicates a moderate to large effect. Accordingly, PASS 15.0 software was used to estimate the required sample size, assuming a significance level (α) of 0.05, a power of 0.80, a two-sided *t*-test with unequal variances, and a 2:1 group allocation ratio. After accounting for a 20% potential dropout or loss to follow-up, the final estimated sample size was 131 (87 in the survival group and 44 in the death group). The actual cohort exceeded this threshold, ensuring adequate statistical power.

### 2.6. Data Collection and Clinical Scores

The following data were collected at the time of enrolment: (1) demographic information (age, sex, hypertension, diabetes mellitus, smoking, alcohol consumption); (2) laboratory data: all the participants fasted for 8 to 12 h. The following morning, fasting venous blood samples were obtained and sent to the hospital’s laboratory department within two hours for examination. Parameters included white blood cell count (WBC), neutrophils, monocytes, platelets (PLT), alanine aminotransferase (ALT), aspartate transaminase (AST), alkaline phosphatase (ALP), γ-glutamate aminotransferase (GGT), total bilirubin, creatinine (Cr), international normalized ratio (INR), albumin (ALB); (3) Complications: ascites, infection, and hepatic encephalopathy. Patients were monitored to calculate the SIRI, CTP [43], MELD [44], MELD-Na [45], and CLIF-C OFs [46] on days 0, 3, 7, and 14 after admission.

#### The Systemic Inflammation Response Index (SIRI)

**Figure 1:**
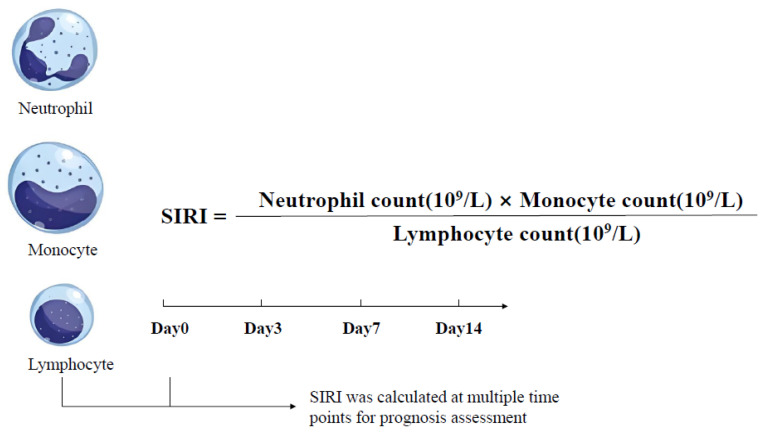
Formula and time points of systemic inflammation response index (SIRI) in HBV-ACLF patients.

The SIRI was introduced as an indicator to assess the inflammatory and immune status of HBV-ACLF patients. It was calculated using the following formula [36] (Figure 1). All parameters in the formula were obtained from routine peripheral blood tests. SIRI values were calculated at baseline (day 0, the day of enrollment), and on days 3, 7, and 14 after admission.

### 2.7. Follow-Up and Outcomes

Patients were followed for 90 days through telephone or hospital records and were subsequently categorized into death or survival groups based on clinical outcomes. Patients who underwent liver transplantation or were lost to follow-up were excluded from the final analysis.

### 2.8. Clinical Definitions

Cirrhosis was diagnosed based on liver biopsy, the evidence of hepatic nodules shown in abdominal imaging, a history of clinical decompensation, or endoscopic evaluation revealing esophageal and gastric variceal bleeding [47].

Smoking was defined as the daily consumption of at least one cigarette daily for a minimum duration of six months [48]. Alcohol consumption was defined as over 40 g/day for men or 20 g/day for women for a period reaching five years [49]. Diabetes was defined by random blood glucose levels ≥ 11.1 mmol/L, fasting blood glucose ≥ 7.0 mmol/L on at least two occasions, HbA1c levels ≥ 6.5%, or a prior diagnosis of diabetes with ongoing use of antidiabetic medications [50]. Hypertension was defined as systolic blood pressure (SBP) ≥ 140 mmHg and/or diastolic blood pressure (DBP) ≥ 90 mmHg on at least three separate days, or a previous diagnosis of hypertension with current use of antihypertensive medications [51].

The virologic breakthrough was defined as a 10-fold increase in serum HBV DNA from the lowest level during treatment in patients who initially responded well to antiviral treatment and did not change their treatment plan. Naïve antiviral therapy was defined as the absence of prior antiretroviral treatment (ART), while antiviral discontinuation indicates the termination of antiviral therapy and consequent viral rebound [52].

Ascites were diagnosed based on clinical manifestations, diagnostic abdominal puncture, and abdominal imaging [53]. Hepatic encephalopathy was diagnosed using the West Haven criteria [54]. Bacterial infections, including spontaneous peritonitis, pulmonary infection, gastrointestinal infection, and sepsis, were diagnosed according to established consensus guidelines [55].

### 2.9. Statistical Analysis

All statistical analyses were performed and figures plotted using R version 4.2 and GraphPad Prism version 10.1.2. Normally distributed continuous variables were presented as the mean ± standard deviation (SD), with group comparisons analyzed using the Student’s *t*-test. Non-normally distributed continuous variables were presented as median (interquartile range (IQR)), and group comparisons were conducted using the Mann–Whitney U test. Categorical variables were presented as frequencies and percentages, with group comparisons analyzed using the chi-square or Fisher’s exact test. Transplantation-free survival at 28, 60, and 90 days was analyzed using Kaplan–Meier analysis. Longitudinal changes in SIRI and disease severity scores (MELD, MELD-Na, CTP, and CLIF-C OFs) at days 0, 3, 7, and 14 were analyzed using linear mixed-effects models, with group, time, and the group × time interaction as fixed effects. The Bonferroni correction was applied for post hoc pairwise comparisons. Univariate and multivariate Cox proportional hazard regression models were used to identify predictors of 90-day mortality. Variables with *p* < 0.05 in the univariate analysis were entered into least absolute shrinkage and selection operator (LASSO) regression with ten-fold cross-validation to select optimal predictors and address multicollinearity. Receiver operating characteristic (ROC) curve analysis was conducted to assess the predictive performance of dynamic changes in SIRI combined with disease severity scores (MELD, MELD-Na, CTP, and CLIF-C OFs) for 90-day mortality. The area under the curve (AUC), sensitivity, and specificity were calculated to assess discriminative ability. *p* < 0.05 was considered statistically significant.

## 3. Results

### 3.1. Baseline Characteristics

**Figure 2:**
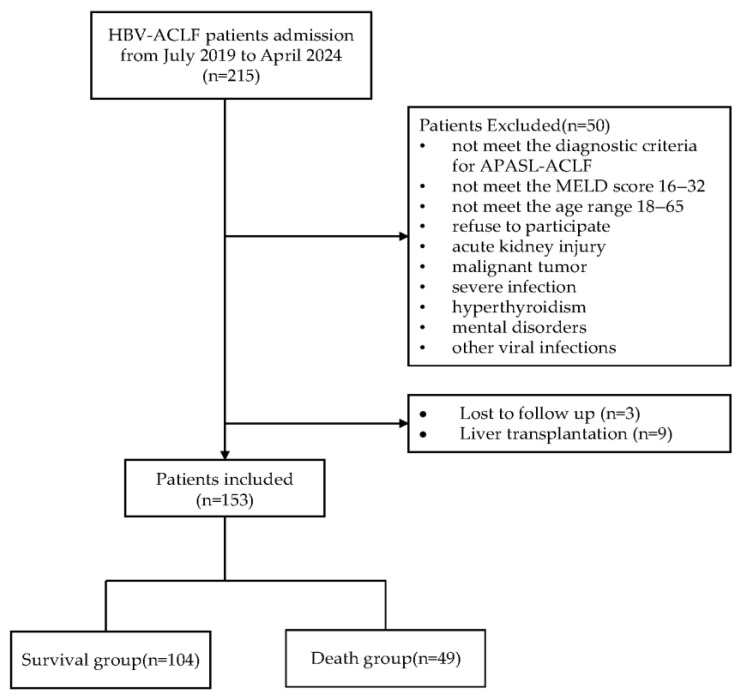
Flowchart for selecting participants.

A total of 215 patients were screened. Of these patients, 50 met at least one exclusion criterion: 11 did not fulfill the APASL-ACLF criteria, 8 did not meet the MELD score 16–32 range, 7 refused to participate, 6 presented with AKI, 5 did not meet the age range, 5 had malignant tumors, 4 had uncontrolled infection, 2 had hyperthyroidism, 1 had psychiatric disorders, and 1 was complicated with another virus infection. Ultimately, 165 patients were enrolled. During the follow-up, three patients were lost to follow-up and nine underwent liver transplantation. A total of 153 patients were included in the final analysis. Figure 2 displays the flowchart for selecting participants.

The participants were categorized into two distinct groups—those who survived and those who did not—based on their 90-day outcomes. The survival group comprised 104 individuals, including 86 men and 18 women. Conversely, the death group consisted of 49 cases, with 45 men and 4 women. The baseline differences between the two groups in terms of co-morbidities, clinical features, and laboratory parameters were analyzed. Notably, the death group exhibited significantly higher levels of ALP, Cr, INR, total bilirubin, MELD, MELD-Na, CTP, and CLIF-C OFs scores compared to the survival group (*p* < 0.05, Table 1). SIRI values at days 0, 3, 7, and 14 were significantly higher in the death group than in the survival group, with all comparisons showing statistically significant differences (Table 2). Additionally, patients were stratified into high and low SIRI groups based on the median SIRI0 value (1.944), and comparisons of baseline characteristics between groups are summarized in Supplementary Table S1.

### 3.2. Prognostic Outcomes and Dynamic Inflammatory Profiles

**Figure 3:**
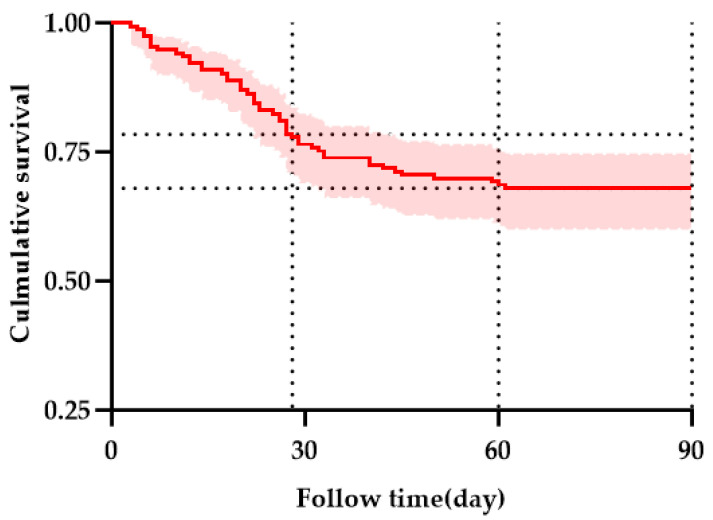
Kaplan–Meier curves showing the survival of all enrolled patients. Horizontal reference lines indicate TFS at 28 and 60 days. The red line with a shaded area indicating the 95% confidence interval.

Figure 3 presents the Kaplan–Meier (KM) survival curve for the entire group, illustrating transplantation-free survival over time. At 28 days, the transplantation-free survival was 78.43%, with 120 patients surviving and 33 patients dying. At 60 days, the transplantation-free survival was 67.97%, with 104 patients surviving and 49 patients dying. At 90 days, the transplantation-free survival was 67.97%, with 104 patients surviving and 49 patients dying.

**Figure 4:**
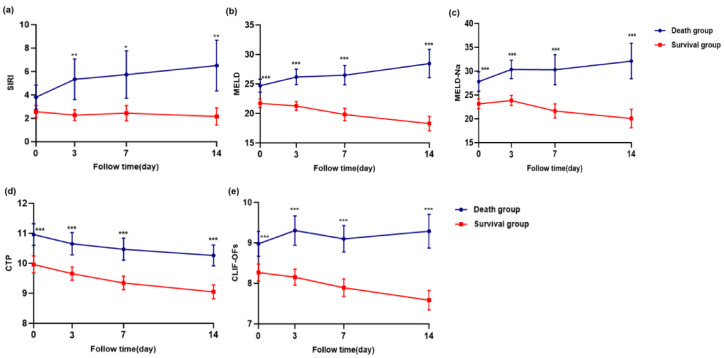
Longitudinal changes in SIRI and disease severity scores (MELD, MELD-Na, CTP, and CLIF-C OFs) at days 0, 3, 7, and 14 in the survival and death groups of patients with HBV-ACLF. Panels (**a**) SIRI, (**b**) MELD, (**c**) MELD-Na, (**d**) CTP, (**e**) CLIF-C OFs. Statistical comparisons were performed using linear mixed-effects models for repeated measures, including group, time, and group × time interaction as fixed effects. Bonferroni correction was applied for post hoc pairwise comparisons. *p* < 0.05, <0.01 and <0.001 were considered statistically significant and are indicated by one (“*”), two (“**“), and three (“***”) asterisks, respectively.

Longitudinal changes in SIRI, MELD, MELD-Na, CTP, and CLIF-C OF scores at days 0, 3, 7, and 14 were compared between the survival and death groups using linear mixed-effects models. As shown in Figure 4a–e, SIRI and disease severity scores were significantly higher in the death group (*p* < 0.05).

### 3.3. Identification of Prognostic Factors via Univariate Analysis and LASSO-Selected Multivariate Cox Regression

**Figure 5:**
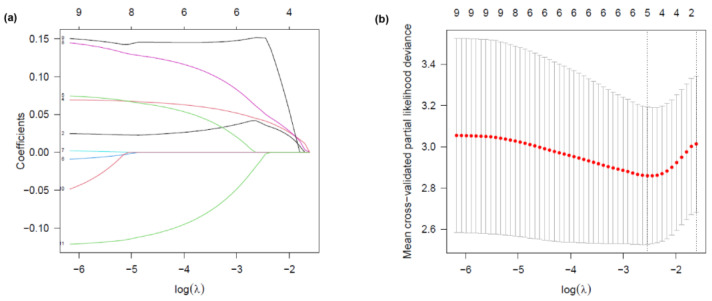
LASSO regression analysis for variable selection. (**a**) LASSO coefficient profile plot. Each colored line represents the coefficient trajectory of a candidate variable as a function of log(λ). Variables with non-zero coefficients at the optimal λ were selected for the model. (**b**) Selection of the tuning parameter (λ) in the LASSO Cox regression model using ten-fold cross-validation. Partial likelihood deviance is plotted against log(λ). Dotted vertical lines indicate the optimal lambda that minimizes the deviance, and the most regularized model within one standard error. Six variables with non-zero coefficients were selected at the optimal λ.

Univariate Cox regression analysis identified SIRI0, SIRI3, SIRI7, SIRI14, ΔSIRI3, ΔSIRI7, and ΔSIRI14, along with MELD, CTP, and CLIF-OFs, to be significantly associated with 90-day prognosis in patients with HBV-ACLF (*p* < 0.05, Table 3). To further identify independent prognostic factors and reduce potential multicollinearity, LASSO regression was applied to variables with *p* < 0.05 in the univariate Cox analysis. Ten-fold cross-validation was used to select the optimal lambda value (λ = 0.0257), minimizing the mean cross-validated partial likelihood deviance. The coefficient path plot revealed that six variables retained non-zero coefficients at this λ value: SIRI3, SIRI14, ΔSIRI3, MELD, CTP, and ALB (Figure 5). Considering collinearity between baseline and dynamic SIRI values and the clinical relevance, only ΔSIRI on day 3 (ΔSIRI3) was retained for the multivariate Cox regression model. Consequently, ΔSIRI3, MELD, CTP, and ALB were included in the final multivariate model.

Multivariate Cox regression revealed that increased ΔSIRI at day 3 (ΔSIRI3), a higher MELD score, and lower albumin levels were independently associated with increased 90-day mortality. The CTP score showed no significant association (Table 4).

### 3.4. Predictive Value of SIRI Combined with Disease Severity Score for 90-Day Prognosis in HBV-ACLF Patients

**Figure 6:**
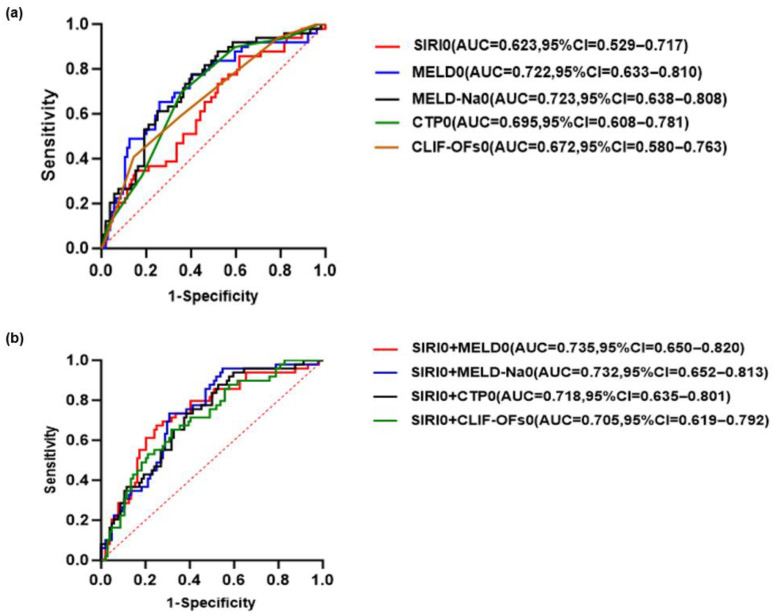

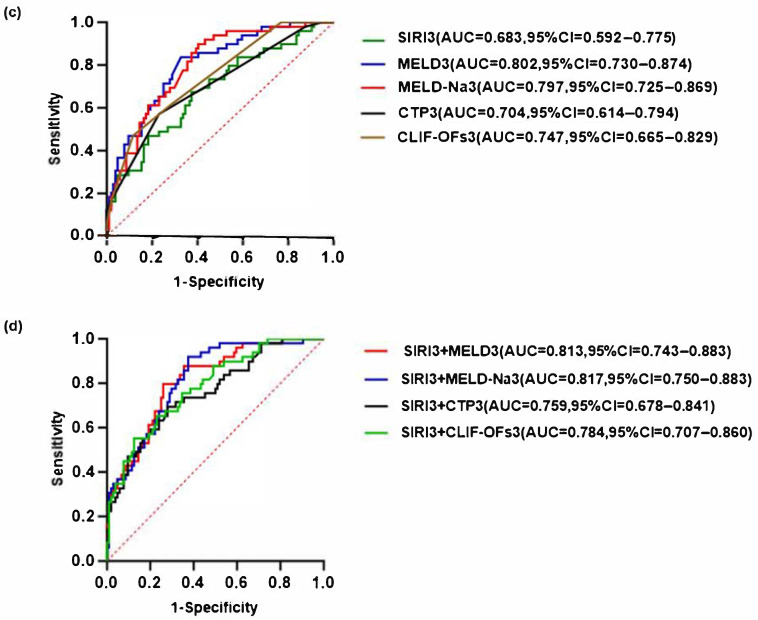
Receiver operating characteristic (ROC) curves for predicting the 90-day prognosis of patients with HBV-ACLF (**a**) SIRI and disease severity scores at baseline predict the prognosis of HBV-ACLF. (**b**) The predictive value of SIRI combined with various disease severity scores at baseline for the prognosis of HBV-ACLF. (**c**) SIRI and disease severity score at day 3 predict the prognosis of HBV-ACLF. (**d**) The predictive value of SIRI combined with various disease severity scores at day 3 for the prognosis of HBV-ACLF. The red dashed diagonal line represents the reference line indicating no discrimination.

The receiver operating characteristic (ROC) curve was generated to evaluate the predictive performance of SIRI combined with various disease severity scores (MELD, MELD-Na, CTP, CLIF-C OFs) for the 90-day prognosis of HBV-ACLF at baseline and day 3, respectively. Among them, the combination of SIRI on day 3 (SIRI3) and MELD-Na on day 3 (MELD-Na3) had the highest AUC, with a sensitivity of 91.84% and a specificity of 62.50%, exceeding that of the baseline combined disease severity score (Figure 6).

## 4. Discussion

Hepatitis B virus (HBV) infection is a major global health burden, with approximately one million deaths annually resulting from acute or chronic HBV infection [56]. ACLF is characterized by rapid deterioration and high short-term mortality, with limited effective medical treatment. Therefore, early disease evaluation and prognostic assessment are essential for timely clinical decision making [57]. In this study, we focused on identifying early prognostic indicators in HBV-ACLF patients. To avoid confounding effects on survival outcomes, patients who underwent liver transplants or were lost to follow-up were excluded from prognostic analysis. Liver transplantation, as the most effective treatment for ACLF, may distort the natural course of the disease. Furthermore, because liver transplantation is not randomly assigned, confounding factors including the affordability of liver resources and transplant indications could be relevant.

At baseline, the proportion of virological breakthroughs in the death group was significantly higher than that in the survival group. Although not statistically significant, the percentage of HBeAg-positive patients in the death group were higher than those in the survival group. Interruption of or reduction in nucleos(t)ide analogues (NAs), or drug resistance, may lead to viral rebound and subsequent virological or biochemical breakthroughs, leading to massive hepatocyte necrosis and ACLF development [58]. In addition, HBV-DNA quantification reflects that the level of viral replication, when excessive, may provoke uncontrolled immune-mediated damage, as is consistent with previous studies [59,60]. These findings highlight the dual role of HBV in ACLF. In some patients, HBV infection leads to sustained viral replication and progressive liver dysfunction. In others, HBV serves as an acute precipitating factor, through virological breakthrough or interruption of antiviral treatment.

Systemic inflammation is a hallmark of ACLF and is strongly correlated with disease severity and poor prognosis [61,62]. Many studies have demonstrated that peripheral blood cell counts and derived ratios can serve as indicators of inflammation and immune function of liver failure [10,63]. As important immune cells, neutrophils, monocytes, and lymphocytes play critical roles in the progression of ACLF [64,65]. When ACLF occurs, a significant number of hepatocytes undergo necrosis, releasing DAMPs, which trigger the immune and systemic inflammatory responses [33,66]. Monocytes and neutrophils exhibit chemotactic, phagocytic, and bactericidal roles, and elevated levels reflect enhanced responsiveness to inflammation [67]. Moreover, excessive immune activation impairs lymphocyte production and cell death, leading to a decrease in lymphocyte count, representing immune imbalance and reduced surveillance [68,69,70].

Qi et al. characterized the SIRI value as a novel marker of inflammation [36]. Compared with single inflammatory markers, SIRI integrates monocyte, neutrophil, and lymphocyte count, providing a simple, readily available index that more comprehensively reflects the inflammatory and immune status. It has demonstrated prognostic value in malignancies such as hepatocellular, colorectal, and gallbladder carcinomas [71,72], as well as in cardiovascular conditions such as hypertension and heart failure [73,74]. In decompensated cirrhosis patients, elevated SIRI has been associated with poorer short-term survival [75], and a recent study of 149 HBV-ACLF patients reported that higher SIRI was correlated with more severe liver injury [76]. However, the prognostic significance of dynamic changes in SIRI during ACLF progression has not yet been explored.

This study investigated the influence of dynamic changes in SIRI on HBV-ACLF prognostic value. Longitudinal data on SIRI and disease severity scores (MELD, MELD-Na, CTP, CLIF-OFs) were analyzed using linear mixed-effects models for repeated measures, which allowed for a robust evaluation of within-subject changes over time while accounting for inter-individual variability. We observed dynamic SIRI value shifts in ACLF progression, with the survival group patients exhibiting lower SIRI levels than the death group on days 0, 3, 7, and 14. A similar trend was observed for disease severity scores. Multivariate Cox regression analysis confirmed that the change in SIRI from baseline to day 3 (ΔSIRI3) was an independent predictor of 90-day mortality. This underscores the critical importance of early inflammatory trajectory in ACLF prognosis. The 72-h window appears to represent a pivotal phase in disease evolution, during which immune dysregulation may either stabilize or worsen. A rising ΔSIRI3 may indicate a suboptimal therapeutic response or the progression of occult infections, warranting closer monitoring and potential escalation of care.

While ΔSIRI3 was identified as an independent prognostic factor in multivariate Cox regression, absolute SIRI values on day 3 (SIRI3) were used in ROC analysis and predictive modeling due to their greater clinical feasibility and interpretability. Importantly, the combination of SIRI3 with MELD-Na3 significantly enhanced prognostic accuracy (AUC = 0.817), supporting dynamic multi-parameter assessment during the early phase of hospitalization. The high sensitivity (91.84%) ensures most high-risk patients are identified, while moderate specificity (62.50%) reflects the multifactorial complexity of ACLF. The complementary use of both dynamic change (ΔSIRI3) and absolute value (SIRI3) enables a more practical assessment of prognosis, supporting both statistical robustness and applicability in HBV-ACLF management. This balance supports its potential utility as a screening tool to prioritize intensive monitoring or early therapeutic interventions.

In contrast, ΔSIRI7 and ΔSIRI14 were not independently predictive, possibly due to treatment-related modulation of inflammation after day 3, reduced statistical power from early mortality, or stabilization of immune responses beyond the acute phase. Furthermore, in the Cox regression, proportion of HBeAg positive results were not statistically significant, while ΔSIRI3 values remained independently associated with 90-day mortality, considering that, although HBeAg status are critical indicators of viral activity, their prognostic value in HBV-ACLF remains complicated. This suggests that systemic inflammatory markers were more predictive of 90-day mortality than virological markers. This is consistent with previous findings suggesting that host immunological and inflammatory responses, rather than viral replication alone, drive disease progression in ACLF [30,77]. While age did not reach statistical significance in association with prognosis in this cohort (*p* = 0.083), a marginal trend was observed. We acknowledge that the pre-specified age range (18–65 years) is a notable limitation, particularly given the established clinical importance of age in HBV-ACLF progression and its potential complex interactions with systemic inflammation. This age restriction may limit the generalizability of our findings, especially to older patients. To address this limitation and better elucidate the prognostic role of SIRI across the age spectrum, future studies should incorporate age-stratified analyses.

It is worth noting that baseline bacterial infections, although clinically controlled, were more common in the death group. Bacterial infections can induce systemic inflammatory responses and affect peripheral immune cell populations, which may in turn influence composite inflammatory indices such as SIRI. However, unlike other indices (e.g., SII) that are significantly impacted by concurrent reductions in platelet and lymphocyte counts, SIRI combines neutrophils, monocytes, and lymphocytes, which may provide a more stable reflection of systemic inflammation in such conditions. This suggests that SIRI may retain prognostic validity even in the presence of low-grade infection.

In addition, the 90-day transplantation-free survival rate observed in this study was 68%, consistent with prior research indicating that ACLF patients enrolled based on APASL criteria had 90-day survival rates between 60% and 70% [78,79,80]. The diagnostic criteria of APASL emphasize liver and coagulation failure, in contrast to the EASL-ACLF criteria, which concentrate on systemic organ failure. ACLF induced by viruses is marked by significant hepatocyte necrosis, systemic inflammatory response, and subsequent complications such as secondary infections, and renal injury, ultimately resulting in poor prognosis [81,82].

Various liver function severity scores, including MELD, MELD-Na, CTP, and CLIF-OFs, can effectively identify the severity of ACLF and predict prognosis [83,84,85]. However, due to the multifactorial etiology of ACLF, using a single indicator to evaluate prognosis has certain limitations. A study reported that the MELD score exhibits limited sensitivity and specificity for ACLF diagnosis and does not incorporate inflammation-related indicators [86]. Our research revealed ROC analyses indicating that the prognostic predictive accuracy for ACLF patients can be improved by combining SIRI3 with the disease severity score. Notably, the exclusion of MELD > 32 and Grade III-IV HE patients is consistent with the natural history of high-risk ACLF. The international guidelines explicitly indicate that these patients should be prioritized for transplant evaluation above simply receiving pharmaceutical therapies [41,87]. In guiding treatment, particularly for HBV-ACLF, the change in SIRI values can be monitored dynamically. When the SIRI value continues to rise during the treatment process, integrated with the MELD-Na score, the treatment strategy can be adjusted over time, incorporating artificial liver support, antiviral medications, or liver transplantation to inform clinical management to improve short-term prognosis.

This study has several limitations. First, the relatively small sample size may limit generalizability and introduce bias. Although post hoc power analysis based on observed data yielded a moderate-to-large effect size (Cohen’s d ≈ 0.67), and the sample size exceeded the estimated requirement, future studies should incorporate more rigorous sample size estimation methods during study design and include larger, independent cohorts to validate these results. Second, SIRI may be affected by treatment protocols. Although all patients received standardized supportive care, individual therapeutic variations were not explicitly analyzed. Future studies should incorporate stratified analysis based on treatment regimens to determine whether SIRI remains a robust prognostic marker across different interventions. Third, the changes in additional inflammatory markers, including CRP and PCT, were not evaluated during the treatment. Fourth, limiting the cohort to ages of 18–65 years minimizes confounding from prevalent age-related comorbidities and polypharmacy, enhancing internal validity for the SIRI-prognosis association. However, this severely restricts generalizability and direct applicability to elderly patients (>65 years), a crucial real-world HBV-ACLF population. Future studies must prioritize including older patients to validate the prognostic utility of SIRI and explore age-specific thresholds. Fifth, our conclusions are applicable only to ACLF patients with MELD 16–32. Since we excluded patients with MELD scores > 32 and those with grade III–IV hepatic encephalopathy, the prognostic value of SIRI in high-risk populations (e.g., MELD > 32 or Grade III-IV HE) requires further validation in future studies. Sixth, given the study design, we did not continually collect long-term follow-up data over 90 days. Future prospective studies are necessary to assess the prognostic significance of SIRI in long-term follow-up. Seventh, some variables with high missing rates, such as HBV DNA, were excluded from the study, and missing data were acknowledged as an additional limitation. Lastly, although patients with uncontrolled infections were excluded, those with controlled infections were included to reflect real-world clinical practice. Since bacterial infections may affect SIRI, future studies with larger cohorts should adjust for infection status in prognostic modeling.

## 5. Conclusions

In conclusion, this study highlights the prognostic significance of the systemic inflammation response index (SIRI) measured on day 3 in HBV-ACLF patients (MELD 16–32). As an easily obtainable and clinically applicable biomarker, SIRI on day 3 notably enhances the predictive accuracy when combined with the MELD-Na score. Therefore, dynamic monitoring of SIRI, especially on the third day, has important clinical guiding value for evaluating the efficacy of existing treatments and deciding whether additional interventions are needed to alleviate liver failure.

## Figures and Tables

**Table 1:** Baseline demographic and clinical characteristics of survival and death groups.

Characteristics	Total (*n* = 153)	Survival Group (*n* = 104)	Death Group (*n* = 49)	Statistic (Z/t/X^2^)	*p*
General					
Age (years)	47.44 (38.81, 54.56)	46.67 (37.63, 53.34)	49.80 (40.82, 57.47)	−1.61	0.107
Sex, male (%)	131 (85.62)	86 (82.69)	45 (91.84)	2.26	0.133
Cirrhosis (%)	29 (18.95)	17 (16.35)	12 (24.49)	1.44	0.230
Smoking status (%)	53 (34.64)	35 (33.65)	18 (36.73)	0.14	0.709
Alcohol consumption (%)	42 (27.45)	29 (27.88)	13 (26.53)	0.03	0.861
Diabetes (%)	11 (7.19)	5 (4.81)	6 (12.24)	1.76	0.185
Hypertension (%)	12 (7.84)	6 (5.77)	6 (12.24)	1.14	0.286
Precipitating events					
Naïve antiviral therapy (%)	81 (52.90)	61 (58.70)	20 (40.80)	4.25	0.039
Antiviral discontinuation (%)	21 (13.70)	11 (10.60)	10 (20.40)	2.72	0.099
Virological breakthrough (%)	36 (23.50)	20 (19.20)	16 (32.70)	3.34	0.068
Others (%)	15 (9.80)	12 (11.50)	3 (6.10)	0.58	0.293
Laboratory values					
WBC (10^9^/L)	6.35 (4.82, 8.57)	6.08 (4.68, 8.06)	6.91 (5.29, 8.64)	−1.50	0.135
Neutrophil (10^9^/L)	4.25 (2.85, 6.24)	3.93 (2.83, 6.08)	4.81 (3.78, 6.44)	−1.88	0.060
Monocyte (10^9^/L)	0.50 (0.37, 0.75)	0.49 (0.37, 0.74)	0.60 (0.41, 0.76)	−1.14	0.254
Lymphocyte(10^9^/L)	1.21(0.79, 1.67)	1.24(0.84, 1.80)	1.15(0.67, 1.38)	−1.64	0.102
PLT (10^9^/L)	92.00 (60.00, 132.00)	95.00 (61.50, 132.25)	82.00 (58.00, 118.00)	−1.39	0.164
ALT (IU/L)	227.60 (75.00, 639.30)	196.00 (70.00, 646.47)	276.00 (98.00, 633.00)	−0.95	0.341
AST (IU/L)	240.00 (104.00, 548.00)	208.60 (95.75, 511.03)	328.00 (151.00, 602.00)	−1.80	0.072
GGT (IU/L)	86.00 (55.00, 125.00)	83.15 (54.50, 114.50)	95.70 (59.40, 132.00)	−1.12	0.265
Total bilirubin (mmol/L)	287.40 (205.60, 400.10)	269.20 (172.78, 349.88)	343.70 (255.90, 464.50)	−3.64	<0.001
Cr (mmol/L)	70.00 (60.80, 82.60)	67.90 (60.00, 78.25)	77.00 (65.00, 90.00)	−2.53	0.011
INR	1.96 (1.69, 2.33)	1.87 (1.67, 2.26)	2.22 (1.80, 2.51)	−2.77	0.006
ALB (g/L)	30.45 ± 4.28	30.96 ± 4.11	29.36 ± 4.47	2.17	0.031
HBeAg positive (%)	86 (56.21)	56 (53.85)	30 (61.22)	0.74	0.391
Disease severity					
MELD score	22.47 (19.88, 25.70)	21.51 (19.14, 23.79)	25.05 (22.42, 26.95)	−4.41	<0.001
MELD-Na score	23.60 (20.41, 27.23)	22.50 (19.50, 25.97)	26.64 (23.45, 30.81)	−4.44	<0.001
CTP score	10.00 (9.00, 11.00)	10.00 (9.00, 11.00)	11.00 (10.00, 12.00)	−3.95	<0.001
CLIF-C OFs score	8.00 (8.00, 9.00)	8.00 (8.00, 9.00)	9.00 (8.00, 10.00)	−3.57	<0.001
Complications of cirrhosis					
Ascites (%)	107 (69.93)	68 (65.38)	39 (79.59)	3.20	0.074
Hepatic encephalopathy (%)	14 (9.15)	7 (6.73)	7 (14.29)	1.47	0.226
Bacterial infection ^a^ (%)	46 (30.07)	23 (22.12)	23 (46.94)	9.76	<0.001

Data are presented as *n* (%) for categorical variables and median (interquartile range) or mean ± standard deviation for continuous variables. According to the APASL criteria, the presence of ascites and/or hepatic encephalopathy (HE) within one month prior to enrollment fulfills the diagnostic requirement for ACLF. Of the 153 patients, 107 had ascites and 14 had HE. The remaining 32 patients (20.9%) had their ascites or HE alleviated before enrollment due to initial treatment or referral, but the diagnosis was confirmed based on prior medical records. Thus, all patients fulfilled the criterion “development of ascitic or hepatic encephalopathy within a month”. Comparisons between survival and death groups were performed using the Chi-square test or Fisher’s exact test for categorical variables, and a Mann–Whitney U test or independent *t*-test was used for continuous variables as appropriate. *p*-values < 0.05 were considered statistically significant. ^a^ Infection refers to clinically controlled bacterial infections present at baseline. WBC: white blood cells; PLT: platelets; ALT: alanine aminotransferase; AST: aspartate transaminase; GGT: γ-glutamyl transpeptidase; Cr: creatinine; INR: international normalized ratio; ALB: albumin; MELD, model for end-stage liver disease; MELD-Na, MELD-sodium; CTP: Child–Turcotte–Pugh score; CLIF-C OFs, chronic liver failure consortium organ failure.

**Table 2:** Comparison of SIRI values between survival and death groups at different timepoints.

Characteristics	Total (*n* = 153)	Survival Group (*n* = 104)	Death Group (*n* = 49)	Statistic (Z)	*p*
SIRI0	1.94 (0.96, 3.96)	1.76 (0.91, 3.45)	2.29 (1.40, 5.13)	−2.45	0.014
SIRI3	1.75 (0.84, 3.81)	1.50 (0.79, 3.10)	2.88 (1.47, 6.98)	−3.65	<0.001
SIRI7	1.65 (0.86, 3.75)	1.49 (0.70, 3.04)	3.07 (1.58, 6.11)	−4.22	<0.001
SIRI14	1.29 (0.80, 3.68)	1.06 (0.56, 1.77)	3.70 (1.75, 6.56)	−6.15	<0.001

SIRI: systemic immune inflammation index. Data are presented as the median (interquartile range). Group comparisons between the survival and death groups at each time point (days 0, 3, 7, and 14) were performed using the Mann–Whitney U test, as the data exhibited a non-normal distribution. SIRI: systemic immune inflammation index.

**Table 3:** Univariate COX analysis of influencing 90-day prognosis in patients with HBV-ACLF.

Variables	Univariate Analysis				
	β	S.E	Z	*p*	HR (95%CI)
Age	0.026	0.015	1.731	0.083	1.026 (0.997–1.057)
Sex	−0.751	0.522	−1.439	0.150	0.472 (0.170–1.312)
WBC	0.072	0.049	1.477	0.140	1.075 (0.977–1.182)
SIRI0	0.084	0.036	2.343	0.019	1.088 (1.014–1.167)
SIRI3	0.096	0.021	4.666	<0.001	1.101 (1.057–1.146)
SIRI7	0.066	0.016	4.112	<0.001	1.068 (1.035–1.102)
SIRI14	0.072	0.015	4.772	<0.001	1.075 (1.043–1.107)
ΔSIRI3	0.092	0.028	3.266	0.001	1.096 (1.037–1.159)
ΔSIRI7	0.072	0.023	3.171	0.002	1.075 (1.028–1.124)
ΔSIRI14	0.067	0.018	3.674	<0.001	1.069 (1.032–1.107)
PLT	−0.003	0.003	−1.114	0.265	0.997 (0.991–1.002)
ALT	0.000	0.000	0.301	0.763	1.000 (1.000–1.001)
AST	0.000	0.000	1.058	0.290	1.000 (1.000–1.001)
GGT	0.001	0.002	0.639	0.523	1.001 (0.998–1.004)
ALB	−0.089	0.037	−2.422	0.015	0.915 (0.851–0.983)
MELD	0.164	0.036	4.573	<0.001	1.178 (1.098–1.264)
CTP	0.416	0.105	3.967	<0.001	1.516 (1.234–1.862)
CLIF-OFs	0.484	0.128	3.789	<0.001	1.623 (1.263–2.084)
Cirrhosis	0.424	0.332	1.275	0.202	1.527 (0.796–2.930)
HBeAg positive (%)	0.216	0.293	0.738	0.461	1.242 (0.699–2.206)

HR: Hazard ratio; CI: confidence interval; WBC: white blood cells; SIRI: systemic immune inflammation index; ΔSIRI3 = SIRI at day 3–SIRI at baseline; ΔSIRI7 = SIRI at day 7–SIRI at baseline; ΔSIRI14 = SIRI at day 14–SIRI at baseline; PLT: platelets; ALT: alanine aminotransferase; AST: aspartate transaminase; GGT: γ-glutamyl transpeptidase; ALB: albumin; MELD, model for end-stage liver disease; CTP: Child–Turcotte–Pugh score; CLIF-C OFs, chronic liver failure consortium organ failure.

**Table 4:** Multivariate COX regression analysis of selected biomarkers in patients with HBV-ACLF.

Variables	Multivariate Analysis				
	β	S.E	Z	*p*	HR (95%CI)
ΔSIRI3	0.107	0.030	3.624	0.001	1.113 (1.051–1.180)
ALB	−0.127	0.052	−2.452	0.014	0.881 (0.796–0.975)
MELD	0.160	0.043	3.711	<0.001	1.174 (1.078–1.277)
CTP	0.061	0.144	0.423	0.672	1.063 (0.801–1.411)

HR: hazard ratio; CI: confidence interval; ΔSIRI3 = SIRI at day 3–SIRI at baseline; ALB: albumin; MELD: model for end-stage liver disease; CTP: Child–Turcotte–Pugh score. Only variables selected through LASSO and retained based on clinical relevance were entered into the model.

## Data Availability

The raw data supporting the conclusions of this article will be made available by the authors on request.

## Supplementary Materials

The following supporting information can be downloaded at: https://www.mdpi.com/article/10.3390/healthcare13172199/s1, Table S1: Comparison of baseline clinical characteristics between high and low SIRI groups in HBV-ACLF patients.

## Abbreviations

The following abbreviations are used in this manuscript:

SIRI	systemic inflammation response index
HBV-ACLF	hepatitis B virus-related acute-on-chronic liver failure
AUC	area under the curve
CTP	Child–Turcotte–Pugh
MELD	model for end-stage liver disease
MELD-Na	model for end-stage liver disease combined with serum sodium
CLIF-C OFs	chronic liver failure combined with organ failure score
APASL	the Asia Pacific Association for the Study of the Liver
AKI	acute kidney injury
WBC	white blood cell count
PLT	platelets
ALT	alanine aminotransferase
AST	aspartate transaminase
ALP	alkaline phosphatase
GGT	γ glutamate aminotransferase
Cr	total bilirubin, creatinine
INR	international normalized ratio
ALB	albumin
SD	standard deviation
IQR	interquartile range
ROC	receiver operating characteristic curves
MT	mechanical thrombectomy
OS	overall survival
HCC	hepatocellular carcinoma
TFS	transplantation-free survival
