# Supplementary material for: The Predictive Role of the Systemic Inflammation Response Index in the Prognosis of Hepatitis B Virus-Related Acute-on-Chronic Liver Failure: A Multicenter Study
Source: Healthcare (Basel). 2025 Sep 2;13(17):2199. doi: 10.3390/healthcare13172199 (PMC12428282; doi:10.3390/healthcare13172199)
Supplement: Supplementary file 1 [file healthcare-13-02199-s001.zip › healthcare-3567050-supplementary.pdf]

Supplementary Table S1. Comparison of baseline clinical characteristics between high and low SIRI groups in HBV-ACLF patients.

Characteristic	Total (n = 153)	Low SIRI Group (n = 76)	High SIRI Group (n = 77)	Statistic (Z/t/X^2)	p
General					
Age (years)	47.44 (38.81, 54.56)	47.34 (36.68, 56.11)	47.44 (40.31, 53.95)	-0.45	0.650
Sex, male (%)	131 (85.62)	63 (82.89)	68 (88.31)	0.91	0.340
Cirrhosis (%)	29 (18.95)	16 (21.05)	13 (16.88)	0.43	0.511
Laboratory values					
WBC (10^9/L)	6.35 (4.82, 8.57)	5.16 (3.85, 6.09)	7.96 (6.47, 9.57)	-7.26	<0.001
Neutrophil (10^9/L)	4.25 (2.85, 6.24)	2.86 (2.28, 3.90)	6.03 (4.66, 7.61)	-8.50	<0.001
Monocyte (10^9/L)	0.50 (0.37, 0.75)	0.40 (0.31, 0.53)	0.66 (0.50, 0.92)	-6.43	<0.001
PLT (10^9/L)	92.00 (60.00, 132.00)	84.00 (46.50, 112.75)	97.00 (67.00, 155.00)	-2.52	0.012
Lymphocyte (10^9/L)	1.21 (0.79, 1.67)	1.29 (1.02, 1.81)	1.00 (0.67, 1.48)	-3.42	<0.001
ALT (IU/L)	227.60 (75.00, 639.30)	172.00 (70.00, 431.73)	281.00 (82.00, 780.00)	-2.24	0.025
AST (IU/L)	240.00 (104.00, 548.00)	197.00 (99.00, 351.50)	328.00 (109.00, 666.00)	-1.83	0.067
GGT (IU/L)	86.00 (55.00, 125.00)	83.65 (50.75, 107.55)	95.70 (63.00, 131.00)	-1.45	0.148
Total bilirubin (mmol/L)	287.40 (205.60, 400.10)	229.45 (135.33, 301.02)	354.60 (274.00, 458.20)	-5.86	<0.001
Cr (mmol/L)	70.00 (60.80, 82.60)	68.10 (60.00, 81.93)	72.70 (61.00, 83.00)	-0.87	0.383
INR	1.96 (1.69, 2.33)	1.94 (1.69, 2.27)	1.99 (1.70, 2.51)	-0.92	0.360
ALB (g/L)	30.30 (27.55, 33.55)	29.65 (27.20, 33.20)	31.20 (28.27, 33.88)	-1.65	0.099
HBeAg positive (%)	86 (56.21)	53 (69.74)	33 (42.86)	11.23	<0.001
Complications of cirrhosis					
Ascites, n (%)	107 (69.93)	55 (72.37)	52 (67.53)	0.43	0.514
HE, n (%)	14 (9.15)	5 (6.58)	9 (11.69)	1.20	0.273
Infection, n (%)	46 (30.07)	21 (27.63)	25 (32.47)	0.43	0.514

Data are presented as n (%) for categorical variables and median (interquartile range) for continuous variables. Patients were stratified based on the median SIRI value at baseline (Day 0). Only variables with less than 15% missing values are presented; variables with high missing rates were excluded to ensure accuracy. Comparisons between survival and death groups were performed using the Chi-square test or Fisher's exact test for categorical variables, and Mann-Whitney U test or independent t-test for continuous variables as appropriate. P-values < 0.05 were considered statistically significant. WBC: white blood cells; PLT: platelets; ALT: alanine aminotransferase; AST: aspartate transaminase; GGT: γ-glutamyl transpeptidase; Cr: creatinine; INR: international normalized ratio; ALB: albumin.
